# *Cladosporium* Infection in a Captive Bottlenose Dolphin (*Tursiops truncatus*): A Rare Case Report from Quanzhou, China

**DOI:** 10.3390/ani15243607

**Published:** 2025-12-15

**Authors:** Kai Jiang, Pengyu Zhao, Lin Cheng, Feiyu Zhao, Lan Bi, Bao Li, Xianjing He, Donghua Guo

**Affiliations:** 1 College of Animal Science and Veterinary Medicine, Heilongjiang Bayi Agricultural University, Daqing 163319, China; jk865789845@163.com (K.J.); zhaopengyu_zoey@163.com (P.Z.); flyyu0805@126.com (F.Z.); 15326593221@163.com (L.B.); 19324639086@163.com (B.L.); xianjinghe@126.com (X.H.); 2 All Love Park Ocean Kindom, Quanzhou 362046, China; chenglin860317@126.com

**Keywords:** *Cladosporium*, cetacean, bottlenose dolphin, captive breeding

## Abstract

This case report describes a male bottlenose dolphin (*Tursiops truncatus*) from a republic aquarium in Quanzhou City, Fujian Province, China, in 2024. The dolphin exhibited prolonged vomiting that did not improve despite extended antibiotic treatment, followed by progressive deterioration in physical condition until death. Antemortem biochemical analyses indicated hepatic dysfunction (ALT: 269.8 IU/L, AST: 1357.5 IU/L, LDH: 2913.3 IU/L) and renal impairment (TBIL: 55.84 μmol/L, BUN: 31.93 mmol/L, Cr: 200.2 μmol/L). Necropsy showed atrophy of coronary fat in the heart, hepatomegaly with extensive yellow discoloration, splenomegaly with congestion, diffuse dark-red discoloration of the lungs, renal atrophy, segmental dark-red discoloration of the intestines, and dark-red enlargement of intestinal lymph nodes. Histopathological examination revealed hepatic steatosis with necrosis, extensive pulmonary hemorrhage with foreign bodies in the trachea and alveoli, intestinal necrosis with visible fungus, and congestion and necrosis of intestinal lymph nodes with visible fungus present; the fungus hyphae were periodic acid–Schiff (PAS)-positive. Fungal PCR targeting the fungus internal transcribed spacer (ITS) region identified the intestine fungus as *Cladosporium*. Infection with *Cladosporium* is extremely rare, and this report highlights the potential risks of emerging infectious diseases in marine mammals.

## Simple Summary

This report describes a case of *Cladosporium* infection identified post-mortem in a 1-year-old male bottlenose dolphin (*Tursiops truncatus*) through necropsy, histopathology, and molecular pathology analyses. Fungal infection was observed via histopathological examination, and the genus of *Cladosporium* was identified by molecular methods. *Cladosporium* infection is an extremely rare disease, and this report highlights the potential risks of emerging infectious diseases in marine mammals.

## 1. Introduction

Dolphins, as vital species in the marine ecosystem, have health condition that are closely linked to the quality of the marine environment. In recent years, the global incidence of dolphin strandings and mortalities has increased significantly. A study on marine cetacean strandings in Italy showed that 41.37% of cetaceans tested positive for viral infections, with cetacean Morbillivirus and Herpesvirus being the most common; 42.70% of cetaceans had bacteria isolates, among which *Brucella* spp. were relatively prevalent; 13.45% were positive for *Toxoplasma gondii* infection; and 7.81% tested positive for fungal infections [1]. While research on cetaceans has largely focused on viral and bacterial pathogens, the economic impact and therapeutic challenges associated with fungal infections should not be overlooked, as they are becoming an increasingly important factor threatening the survival of dolphin populations [1,2,3,4]. Some of the pathogens responsible for these infections are zoonotic and can be transmitted to humans and domestic animals, potentially leading to diseases of considerable economic and public health significance.

Nowadays, fungi isolated from different marine mammals include *Aspergillus* spp., *Blastomyces* spp., *Coccidioides* spp., yeast, *Fusarium* spp., and *Histoplasma* spp., among others [5]. Some fungi are part of the normal microbiota of marine mammals or their environment; others are opportunistic pathogens that can cause diseases when the animals’ health deteriorates. Some opportunistic fungi can cause systemic disease accompanied by respiratory, gastrointestinal, or neurological manifestations, thereby increasing morbidity, mortality, and stranding rates in marine animals and posing a substantial threat to those in captivity. Among these pathogens, *Aspergillus* spp. represent the most common cause of fungal infections in marine mammals, with bottlenose dolphins being particularly susceptible and mortality rates reaching up to 62.5% [6]. *Candida* infections have been reported primarily in captive dolphin populations and may lead to gastrointestinal, respiratory, or systemic disease. In captive settings, the prevalence of oral *Candida* infection can reach 70%, posing a significant health risk to dolphins under human care [3]. Fungal diseases have been reported in marine mammals worldwide, and captive individuals appear to be more susceptible to infection. In this report, we conducted a necropsy on a deceased captive bottlenose dolphin, performed histopathological examinations on some organs, and confirmed *Cladosporium* infection through ITS region sequencing.

## 2. Materials and Methods

On 2 November 2024, a one-year-old male bottlenose dolphin died at a republic aquarium in Quanzhou City, Fujian Province, China. A clinical veterinarian performed a necropsy on the deceased dolphin and documented the findings. One sample each of the liver, spleen, lung, kidney, small intestine, large intestine, and mesenteric lymph node were collected, fixed in 10% formalin, and sent to the College of Animal Science and Veterinary Medicine, Heilongjiang Bayi Agricultural University, for routine histopathological evaluation [7]. Selected tissue sections were subjected to Periodic Acid–Schiff (PAS) staining using a commercial PAS Staining Kit (Beyotime, Shanghai, China) to identify potential fungal elements within the tissues. The stained sections were observed and digitally photographed using a Mshot ML31 microscope (Mshot, Guangzhou, China).

Five 10 μm slices of formalin-fixed-paraffin-embedded (FFPE) intestinal tissue were used for molecular identification of the fungal internal transcribed spacer (ITS) region. Fungal DNA was extracted from these tissues using the Paraffin-Embedded Tissue DNA Extraction Kit (TIANGEN, Beijing, China) and then used in a PCR protocol designed to amplify the ITS1 and ITS4 regions of fungi [8]. The PCR products were separated by electrophoresis in 1% agarose gels, stained with ethidium bromide, and examined under ultraviolet light.

PCR products were Sanger-sequenced using the same primer pair at Sangon Biotech (Shanghai, China). A preliminary evaluation of the sequences was performed via a BLAST 2.17.0 search, then the neighbor-joining (NJ) method was reconstructed using MEGA 7.0 software [9].

## 3. Results

### 3.1. Living Environment and Clinical Biochemistry Analysis

The bottle dolphin and its 10-year-old mother had been housed together in a single isolated enclosure with an approximate water volume of 1600 m^3^. The living environment parameters of the bottle dolphin were as follows: salinity 30‰, pH 7.66, nitrite (NO_2_^−^) 0.07 mg/L, nitrate (NO_3_^−^) > 50 mg/L, and ammonia/ammonium (NH_3_/NH_4_^+^) 0.1 mg/L. The water was treated through sand filtration and ozone disinfection.

The dolphin first exhibited vomiting at the age of 3 months, and this symptom persisted until death. During this period, veterinarians administered medications including cefaclor, amoxicillin–clavulanate potassium, and azithromycin to the dolphin by feeding the drugs to its mother. Before the dolphin’s death, clinical biochemistry analysis revealed abnormal liver and renal function, indicating organ dysfunction. The liver function parameters were as follows: alanine aminotransferase (ALT) 269.8 IU/L, aspartate aminotransferase (AST) 1357.5 IU/L, and lactate dehydrogenase (LDH) 2913.3 IU/L. The renal function parameters were: total bilirubin (TBIL) 55.84 μmol/L, blood urea nitrogen (BUN) 31.93 mmol/L, and creatinine (Cr) 200.2 μmol/L.

### 3.2. Post-Mortem Examination

**Figure 1:**
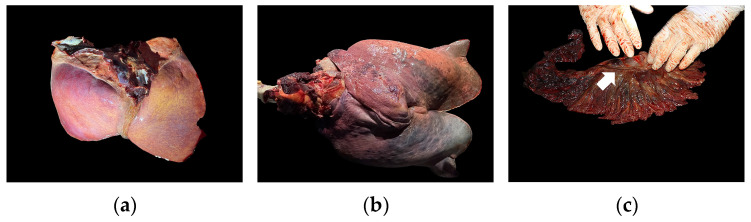
Gross necropsy finding of selected tissues. (**a**) The liver exhibited diffuse yellow discoloration, a fragile texture, and raised margins. (**b**) The lungs showed a diffuse dark-red discoloration. (**c**) The mesenteric lymph nodes were enlarged and dark red in appearance (white arrow).

The deceased bottlenose dolphin was a one-year-old male, measuring 187 cm in length and a weighing 87.2 kg. External examination revealed no obvious trauma on the body surface; however, the skin was covered with black raised particles. A large amount of brownish-yellow effusion was observed within the abdominal cavity. Other gross pathological findings included atrophy of coronary fat in the heart, a yellowish, friable liver (Figure 1a), splenomegaly with congestion, diffuse pulmonary congestion (Figure 1b), renal atrophy, partial exfoliation of the esophageal mucosa, hyperemia, hemorrhage, tympany, and catarrhal inflammation of the intestine, and enlargement and necrosis of the mesenteric lymph nodes (Figure 1c).

### 3.3. Histological and Molecular Pathology Findings

**Figure 2:**
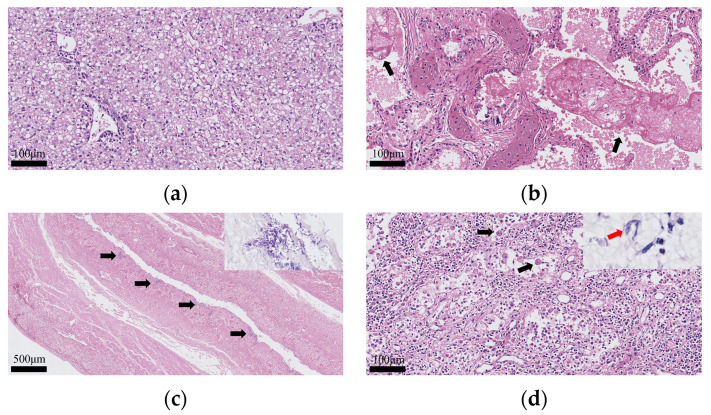
Histopathological examination of selected tissues. (**a**) The liver showing diffuse hepatocellular steatosis, focal lymphocytic infiltration in the portal area, and hepatocyte necrosis. Hematoxylin and eosin (HE) staining; scale bar = 100 μm. (**b**) The lungs showing diffuse hemorrhage in the bronchi and alveoli. Eosinophilic, fibrous foreign material is visible within the bronchi and alveoli (black arrow). HE staining; scale bar = 100 μm. (**c**) The intestine showing mucosal exfoliation and submucosal necrosis. Multifocal fungal colonies with basophilic staining are visible in the mucosal layer (black arrow) with HE staining; scale bar = 500 μm. The fungal colonies appear magenta with PAS staining (inset). (**d**) The mesenteric lymph node showing multiple foci of congestion, lymphocyte necrosis, and medullary edema. Multinucleated giant cells are visible (black arrow) with HE staining; scale bar = 100 μm. The subcapsular sinuses are dilated, and fungal elements appear magenta with PAS staining (red arrow, inset).

Histopathological examination revealed the following pathological changes: the liver exhibited diffuse steatosis accompanied by extensive hepatocellular necrosis, with mild lymphocytic infiltration around the bile ducts in the portal area (Figure 2a); the lung showed marked hemorrhage, and eosinophilic, reticular foreign material was present within the alveoli and bronchioles (Figure 2b); the large intestine segment displayed necrosis and mucosal exfoliation, with numerous fungal aggregates composed of spherical and rod-shaped elements adhering to the intestinal mucosal layer; PAS staining demonstrated positive reactivity of the fungal elements (Figure 2c); the mesenteric lymph nodes exhibited focal necrosis and hemorrhage, accompanied by multinucleated giant cell infiltration (Figure 2d). No significant pathological changes were observed in the spleen, kidneys or small intestine.

**Figure 3:**
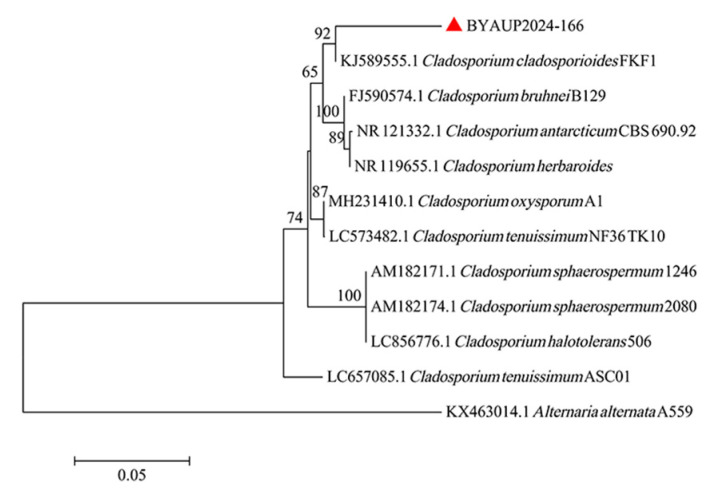
Phylogenetic tree constructed using the neighbor-joining (NJ) method based on fungal ITS rDNA gene sequences. Bootstrap support (>70%) are shown next to the branches. The analysis involved 11 nucleotide sequences, and the Chinese sequence is indicated by a red triangle.

The universal primers successfully amplified a 675-bp fragment of the ITS region from FFPE tissues samples. However, only a high-quality portion of this fragment was used for BLAST analysis. BLAST comparison of the usable partial ITS sequence (GenBank accession no. PX588376) indicated that the fungus belonged to the genus *Cladosporium*, showing the highest similarity (95.23%) to *Cladosporium cladosporioides* (KJ589555.1). The phylogenetic tree of this strain is shown in Figure 3.

## 4. Discussion

This study investigated the death of a captive dolphin that had exhibited recurrent vomiting and received nearly seven months of antibiotic treatment without notable improvement. Initially, the authors and attending veterinarians suspected a *Candida* infection based on the dolphin’s persistent vomiting and irregular exfoliation of the esophageal mucosa [10]. Candidiasis is relatively common in dolphins, with lesions typically involving the skin, oral cavity, gastrointestinal tract, and other organs. Affected skin and mucous membranes often show erosion, ulceration, and pseudomembrane formation, whereas dolphins with gastrointestinal involvement frequently present with vomiting [3,10,11,12]. These clinical signs and pathological findings were largely consistent with those observed in the present case.

Furthermore, the fungal genus was determined through ITS rDNA sequencing. BLAST analysis showed that the ITS rDNA sequence amplified from the genomic DNA extracted from FFPE tissues belonged to the genus *Cladosporium*. Under routine fungal identification procedures, fungal colonies are typically isolated and cultured, followed by morphological examination and ITS rDNA sequencing for species-level determination. However, in this case, improper sample preservation resulted in only FFPE tissues being available, and the limited discriminatory power of the ITS region within this genus prevented accurate species-level identification. To improve diagnostic accuracy, sequencing of additional genetic markers such as translation elongation factor 1-α (*tef1*) and actin (*act*) could provide more reliable species-level resolution [13]. Although definitive species identification was not possible in this case, phylogenetic analysis indicated that the detected sequence clustered most closely with *Cladosporium cladosporioides* (KJ589555.1), offering some reference value regarding its potential species affiliation.

*Cladosporium cladosporioides* is a melanin-containing fungus, and infections caused by pigmented fungi are collectively referred to as “phaeohyphomycosis” [14,15]. *Cladosporium cladosporioides* is commonly found in decomposed organic matter in both outdoor and indoor environments and is recognized as an important food contaminant. Additionally, certain *Cladosporium* species can colonize surfaces such as fiberglass and the interior of water pipes. *Cladosporium cladosporioides* is typically pathogenic to plants and occasionally appears as a contaminant during laboratory cultivation [16,17,18]; it is very rarely reported as an animal pathogen. Documented cases of *C. cladosporioides* infection include those in humans as well as in dogs, cats, sheep, giant pandas, and sea turtles [14,15,16,19,20,21,22,23,24]. The most common presentation involves superficial skin lesions, which may appear as single or multiple erythematous, swollen nodules or ulcers [24]. The lungs are the second-most susceptible site, leading to clinical signs such as dyspnea and coughing in infected animals [20]. Systemic infections have also been reported, including renal involvement in dogs and cerebellum infection in cats [21,23]. Treatment for *C. cladosporioides* infection typically involves oral administration of itraconazole combined with topical ketoconazole, both of which have shown favorable efficacy against superficial and systemic infections without evident hepatotoxic or nephrotoxic effects [14,24,25].

In mammals, *Cladosporium* species are considered opportunistic pathogens capable of inducing cutaneous granulomas in both immunocompromised and immunocompetent hosts, often leading to delayed diagnosis and therapeutic challenges [26,27]. Various factors can impair immune function in animals, including nutritional status, physical condition, and environmental factors such as salinity, temperature, chlorine, and other antimicrobial components [28]. Nitrate (NO_3_^−^) is the most common nitrogen-containing compound in natural aquatic environments and exerts toxic effects on aquatic organisms. As NO_3_^−^ concentrations increase, the cumulative survival rate of juvenile turbot (*Scophthalmus maximus*) decreases in a dose-dependent manner, accompanied by progressively severe hepatic and gill damage [29]. Camargo et al. [30] proposed that concentrations for marine organisms should not exceed 20 mg/L. It is noteworthy that the NO_3_^−^ concentration (>50 mg/L) in the dolphin’s living environment was markedly higher than the recommended safety threshold for marine organisms reported in the literature. Although high nitrate levels have been demonstrated to exert immunosuppressive and organ-toxic effects in fish and may have acted as a potential stressor in this case, it should be emphasized that the specific toxic threshold, mode of action, and direct association between nitrate exposure and increased susceptibility to fungal infection in cetaceans remain unclarified. Therefore, the precise contribution of elevated environmental nitrate to the health condition of this dolphin requires further investigation.

Although the precise pathogenesis of this case remains unclear, it is plausible that *Cladosporium* represented a secondary systemic infection and that the dolphin may have experienced mixed infections prior to death. According to the attending veterinarians, numerous black, raised nodules were present on the skin surface, suggesting the possibility of concurrent viral infection. Cetacean morbillivirus is one of the most frequently reported viral pathogens associated with mortality in cetaceans. Due to its strong tropism for lymphoid tissues, subacute and chronic infections often result in immunosuppression, predisposing animals to secondary opportunistic infections, which may obscure the characteristic clinical signs of morbillivirus-associated disease [31]. Poxvirus infection in dolphins is characterized by irregular gray to black cutaneous lesions of varying size, commonly referred to as “tattoo skin disease.” The prevalence is typically higher in calves and juveniles and tends to decrease in adults [32].

Beyond viral infections, bacterial pathogens may also contribute substantially to mixed infectious processes. *Vibrio alginolyticus* has been aimplicated in fatal bronchopneumonia, septicemia, and meningoencephalitis in dolphins, while *Burkholderia pseudomallei* can induce chronic, diffuse granulomatous inflammation [33,34,35]. Such mixed infections can progressively impair immune function, and prolonged administration of high-dose antibiotics may further disrupt the intestinal microbiota, creating conditions that promote fungal overgrowth. This imbalance could have further damaged the intestinal mucosa and facilitated hematogenous dissemination to internal organs. Although no overt organ lesions were observed in this case, similar fungal infections in other animals have demonstrated such dissemination [20].

## 5. Conclusions

In this study, a fungal infection caused by *Cladosporium* was identified in a bottlenose dolphin through necropsy, histopathology and molecular analysis. This case report indicates that, similar to other animals, dolphins are also susceptible to rare opportunistic fungal diseases. Further research is needed to evaluate the epidemiological significance and potential risk factors associated with such infection in marine mammals. Given that dolphins are sentinel species reflecting the health status of marine ecosystems, understanding these emerging infectious diseases is essential for both animal conservation and marine environmental monitoring.

## Data Availability

The original contributions found in this study are included in this article. Further inquiries can be directed at the corresponding author.

## Abbreviations

The following abbreviations are used in this manuscript:

ALT	Alanine Aminotransferase
AST	Aspartate Aminotransferase
LDH	Lactate Dehydrogenase
TBIL	Total Bilirubin
BUN	Blood Urea Nitrogen
Cr	Creatinine
ITS	Internal Transcribed Spacer
PAS	Periodic Acid–Schiff
PCR	Polymerase Chain Reaction
FFPE	Formalin-Fixed-Paraffin-Embedded
